# Smile analysis in different facial patterns and its correlation with underlying hard tissues

**DOI:** 10.1186/s40510-015-0099-4

**Published:** 2015-09-04

**Authors:** Neha Grover, DN Kapoor, Santosh Verma, Preeti Bharadwaj

**Affiliations:** Nora Medical Polyclinics, Tower 4. Marks and Spencer Building, No. 106, Abu Dhabi, United Arab Emirates; Department of Orthodontics, Kothiwal Dental College & Former Dean of Dental Faculty, KGMU, Lucknow, UP India; Department of Orthodontics, Kothiwal Dental College & Research Centre, Moradabad, UP India; Department of Orthodontics & Dentofacial Orthopedics, KD Dental College & Hospital, Mathura, UP India

## Abstract

**Background:**

The subject’s inherent growth pattern can be an effective factor in characteristics of smile. More vertical growth in the posterior maxilla than in the anterior maxilla could result in a changed relationship between the occlusal plane and the curvature of the lower lip upon smile. In order to broaden the understanding of how smile gets affected by growth pattern and the underlying hard tissues, the present study was undertaken to compare smile in various growth patterns, to determine sexual dimorphism, if any; as well as to correlate smile with underlying hard tissues.

**Methods:**

One hundred and fifty subjects were selected amongst the students in the Dental Institute and from the outpatient department of Department Orthodontics and Dentofacial Orthopedics. Sample selected for the study ranged in the age group of 17 to 25 years. Selected individuals were subjected to lateral head cephalometric radiography in the Department of Oral Medicine and Radiology and videography. Cephalograms were traced and the subjects were divided into horizontal, average, and vertical growth pattern on the basis of GoGn-SN, lower anterior facial height, and Jaraback’s ratio. The video clip was downloaded to obtain frame of posed smile. Cephalometric and photographic measurements were recorded and subjected to statistical analysis.

**Results:**

The mean values of smile parameters were significantly higher in males as compared to females irrespective of the growth pattern. The mean incisal display, interlabial gap, lower lip to incisal edge distance, upper vertical lip length, and occlusal plane angle was highest in both males and females of vertical facial growth pattern group; whereas, the smile index, posterior corridor (left and right) were less in vertical facial growth pattern group in both males and females. Thus, the parameters in vertical dimension were increased in vertical growers whereas, the parameters in transverse dimension decreased.

**Conclusions:**

The facial growth pattern has significant influence on the parameters of smile along with definite sexual dimorphism. The angular and linear parameters, except saddle angle and lower incisor to NB (linear and angular), influenced smile.

**Electronic supplementary material:**

The online version of this article (doi:10.1186/s40510-015-0099-4) contains supplementary material, which is available to authorized users.

## Background

The smile is one of the most important facial expressions and is essential in expressing friendliness, agreement, and appreciation. A smile when pleasing and attractive to observers enriches not only the one who smiles but also those who view it. An attractive or pleasing smile clearly enhances the acceptance of an individual in the society by improving the initial impression in interpersonal relationships [1]. The esthetic considerations are paramount in treatment planning; however, rigid rules cannot be applied to this process because almost an infinite variety of faces could be esthetic [2].

There are two forms of smiles—the enjoyment or Duchenne smile and the posed or social smile. The posed smile is voluntary and not elicited by an emotion. In other words, it is reliably reproducible and can be sustained [3]. Posed smiles, therefore have importance in orthodontic diagnosis and treatment planning. The unposed or social smile, however, is involuntary and is induced by joy or mirth. It is a natural response as it expresses authentic human emotion. Unlike the posed smiles, these smiles are not sustained.

The vertical aspects of smile anatomy are the degree of maxillary anterior tooth display (Morley ratio), upper lip drape, and gingival display. In a youthful smile, 75–100 % of the maxillary central incisors should be positioned below an imaginary line drawn between the commissures [4]. Both skeletal and dental relationships contribute to these smile components. The present study was undertaken to compare smile in various growth patterns, to determine sexual dimorphism, if any; as well as to correlate smile with underlying hard tissues.

## Methods

In this cross-sectional study, a total of 150 subjects were selected amongst the students studying in Dental College and the outpatient department of the College. Selected individuals ranged in age of 17–25 years had class I molar relation, well aligned anterior teeth, presence of all permanent teeth except third molars, no gross facial asymmetry, with no previous orthodontic treatment, and no history of facial trauma, plastic surgery, or orthognathic surgery.

**Fig. 1:**
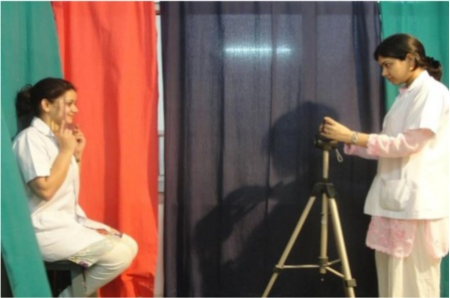
Video camera set on a tripod at 4 ft distance from the subject in sitting position

Selected subjects were first taken for videography using Sony digital camera (DSC-H20, Sony Corp. Japan). Signed consent of the subjects was taken on using video for research purpose. The method used for videography here was as described by Sarver and Ackerman [2, 5, 6]. The subjects were instructed to hold their head in natural head position by looking straight into an imaginary mirror [7]. The camera lens was adjusted parallel to the apparent occlusal plane and focussed only on the dentofacial complex (corresponding to the area from the nose to the chin) (Fig. 1). Included in the captured area were two rulers with millimeter markings. These two rulers were made to fit perpendicular to each other in order to help minimize any error. If the subject was unable to hold the ruler perpendicular in one dimension to the angle in which the dynamic record was taken, the second ruler would still be perpendicular to the camera.

**Fig. 2:**
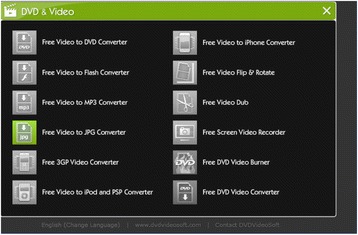
Video to JPEG converter

**Fig. 3:**
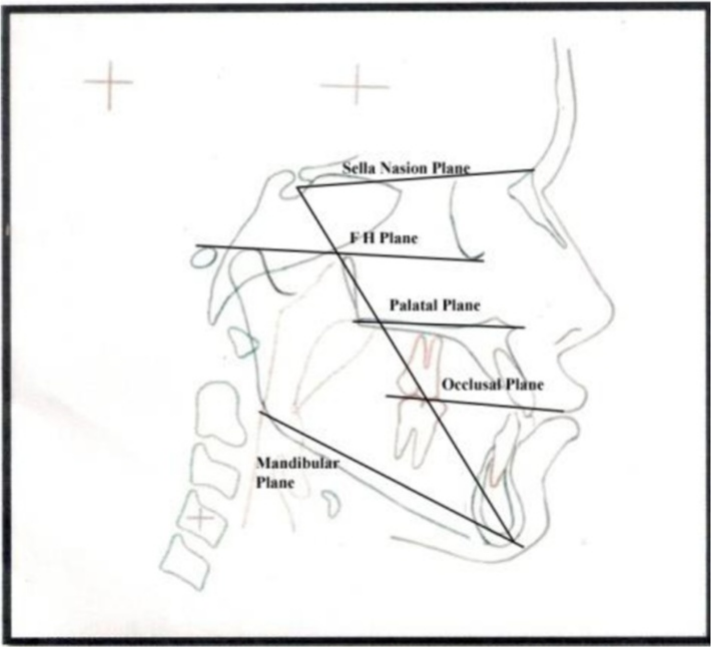
Cephalometric planes

**Fig. 4:**
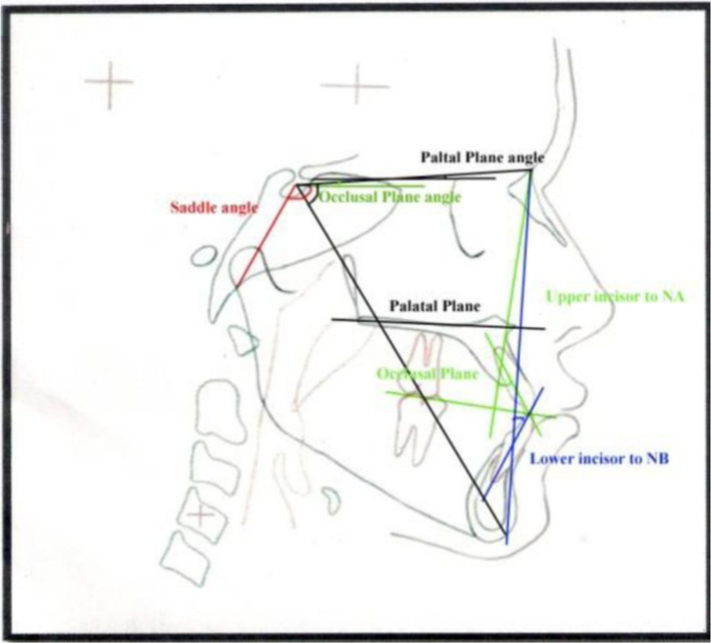
Cephalometric parameters (angular and linear)

Recording was started about 1 s before the subject began to smile and continued till the end of the smile. The video clip was downloaded to laptop (Dell, Inspiron) and uploaded to video-editing software program (DVD VideoSoft Studio) (Figs. 3 and 4) to obtain frames of smile. Each frame was analyzed and the frame showing the subjects’ widest commissure to commissure was chosen as posed smile. These frames were converted into a JPEG file using the same video-editing software program (DVD VideoSoft Studio) (Fig. 2).

The approval was taken from Institute Ethics Committee whose students were involved in the study.

The lateral head cephalogram of the selected subjects were taken with radiograph machine Villa (Italy, Strato 2000) using a standardized technique.

The cephalometric tracings were carried out, and the following cephalometric landmarks and planes were used in the study (Figs. 3 and 4). The parameters used to classify subjects in different groups are shown in Table 1.

**Table 1:** Parameters used for classification of growth pattern

S. no.	Parameters		Average	Vertical	Horizontal
1	Jarabak’s ratio (Jarabak [10])		63.5 ± 1.5 %	<62 %	>65 %
2	SN-GoGn (Steiner [10])		32° ± 2°	>34°	<30°
3	Lower anterior facial height (McNamara [10])	M	72 ± 2 mm	>74 mm	<70 mm
		F	67 ± 1 mm	>68 mm	<66 mm

Based on these three parameters, the subjects were divided into six groups:

A. Group 1: Males with average growth pattern
B. Group 2: Females with average growth pattern
C. Group 3: Males with horizontal growth pattern
D. Group 4: Females with horizontal growth pattern
E. Group 5: Males with vertical growth pattern
F. Group 6: Females with vertical growth pattern

### Standardization of image

**Fig. 5:**
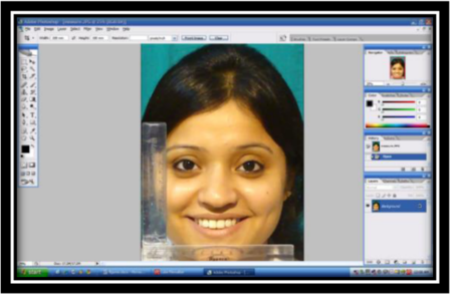
Standardized photograph in Adobe Photoshop

Each JPEG file of the selected subjects were opened in Adobe Photoshop CS2 (Adobe Systems, San Jose, California) and was adjusted by using the ruler option in the frame. The method used to standardize the image was as described by Desai et al [8]. First, the resolution was changed to 300 pixels per inch by going to “image > image size.” Then, the ruler function was chosen and set to millimeter which can measure a minimum of 0.1 mm length. On the parallel end of the ruler, a 10-mm area, close to the smile, was measured. That number was divided into 10 (10/measurement on JPEG file) and multiplied by the width value found in image size screen (image > image size). The resulting number was copied and pasted in place of the width reading, and the changes were applied to the JPEG file (Fig. 5).

In Adobe Photoshop, the following parameters of the selected subjects were measured and entered into Microsoft Excel to evaluate smile:

1. Maximum incisor exposure [9]: the amount of vertical display of the maxillary right central incisor (Fig. 6).

**Fig. 6:**
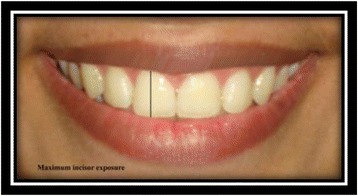
Maximum incisor exposure
2. Lower lip to maxillary incisor [9]: vertical distance from the deepest midline point on the superior margin of the lower lip to the maxillary right central incisor edge (Fig. 7).

**Fig. 7:**
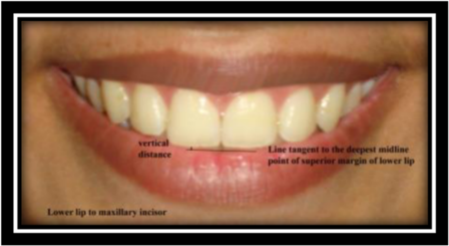
Lower lip to maxillary incisor
3. Interlabial gap [9]: the distance between the most inferior portion of the tubercle of the upper lip and the deepest midline point on the superior margin of the lower lip (Fig. 8).

**Fig. 8:**
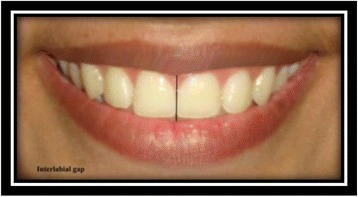
Interlabial gap
4. Maxillary intercanine width [9]: the distance from the distal aspect of the right canine to the distal aspect of the left canine (Fig. 9).

**Fig. 9:**
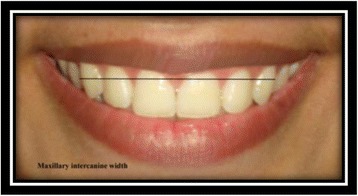
Maxillary intercanine width
5. Width of all visible maxillary teeth [9]: the distance from the distal aspect of the most posterior visible tooth on the right to the most posterior visible tooth on the left side of the maxilla (Fig. 10).

**Fig. 10:**
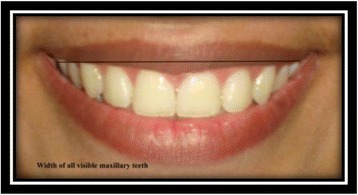
Width of all visible maxillary teeth
6. Smile width [9]: the distance from outer commissure to outer commissure on smile (Fig. 11).

**Fig. 11:**
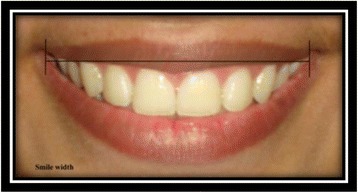
Smile width
7. Smile index [9]: smile width/interlabial gap (Fig. 12).

**Fig. 12:**
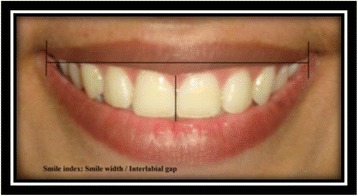
Smile index
8. Right and left buccal corridors [9]: the horizontal distance from the distal aspect of the canine to the respective outer commissure (Fig. 13).

**Fig. 13:**
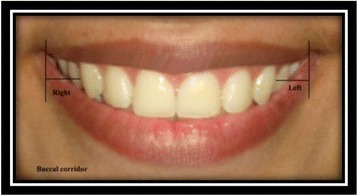
Right and left buccal corridors
9. Right and left posterior corridors [9]: the horizontal distance from the distal aspect of the most posterior tooth visible on smile to the respective outer commissure (Fig. 14).

**Fig. 14:**
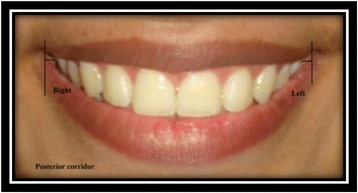
Right and left posterior corridors
10. Buccal corridor ratio [9]: intercanine width/smile width (Fig. 15).

**Fig. 15:**
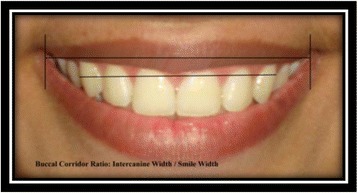
Buccal corridor ratio
11. Posterior corridor ratio [9]: visible maxillary teeth width/smile width (Fig. 16).

**Fig. 16:**
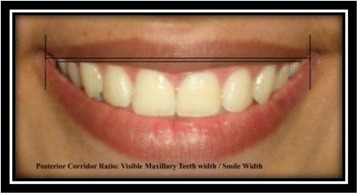
Posterior corridor ratio
12. Upper vertical lip thickness [9]: the vertical distance from the most superior peak of the lip to the most inferior portion of the tubercle of the upper lip (Fig. 17).

**Fig. 17:**
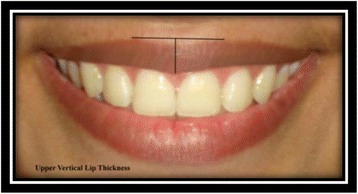
Upper vertical lip thickness
13. Lower vertical lip thickness [9]: the vertical distance from the deepest midline point on the superior margin of the lower lip to the most inferior portion of the lower lip (Fig. 18).

**Fig. 18:**
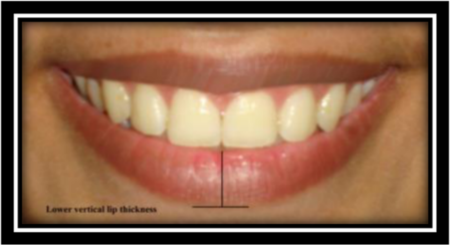
Lower vertical lip thickness

Double determination for assessment of inherent error in measurements was done on 20 random photographs and cephalometric measurements. The variability was found to be statistically insignificant.

## Results

The smile parameters of male and female were compared in all three growth patterns as shown in Tables 2, 3, and 4.

**Table 2:** Comparison of male and female in average growth pattern (comparison of groups 1 and 2)

Parameter	Males	Females	Difference	*t* value	*p* value	Significance
	Mean ± SD	Mean ± SD				
U1 EXP.	8.65 ± 1.04	8.46 ± .85	.192	0.717	.477	NS
LL-U1	.80 ± 0.97	0.14 ± 0.22	.66	3.308	.002	S^*^
IL GAP	9.71 ± 1.64	8.44 ± 0.82	1.276	3.473	.001	S^**^
IC WIDTH	40.29 ± 2.39	39.34 ± 1.95	.952	1.544	.129	NS
TW	53.43 ± 3.88	49.66 ± 3.23	3.768	3.736	<0.001	S^***^
SW	67.39 ± 4.63	65.76 ± 3.42	1.632	1.419	.162	NS
SI	7.16 ± 1.50	7.87 ± 8.87	7.056	−2.02	.049	S^*^
BC_L	13.89 ± 1.542	13.21 ± 1.97	.68	1.358	.181	NS
BC_R	13.66 ± 2.29	12.68 ± 2.01	.98	1.610	.114	NS
PC_L	7.60 ± 2.38	7.99 ± 2.24	−.392	0.60	.552	NS
PC_R	6.82 ± 2.15	7.42 ± 1.95	−.596	1.03	.310	NS
BC RATIO	6.00 ± 5.16	5.99 ± 4.11	6.49	0.049	.961	NS
PC RATIO	7.95 ± 6.71	7.57 ± 6.07	3.832	2.116	.040	S^*^
U VERT	5.98 ± 1.27	5.97 ± 1.49	0.012	0.031	.976	NS
L VERT	11.20 ± 1.03	10.33 ± 1.09	.864	2.884	.006	S^**^

*NS p* > 0.05: insignificant

^*^ *p* < 0.05, significant at 5 % significance level; ^**^ *p* < 0.01, significant at 1 % significance level; ^***^ *p* < 0.001, highly significant

**Table 3:** Comparison of male and female in horizontal growth pattern (comparison of groups 3 and 4)

Parameter	Males (*n* = 25)	Females (*n* = 25)	Difference	*t* value	*p* value	Significance
	Mean ± SD	Mean ± SD				
U1 EXP.	6.93 ± 1.01	7.36 ± 1.14	0.4360	1.43	.160	NS
LL-U1	0.3 ± 0.62	0.15 ± 0.29	0.148	1.081	.285	NS
IL GAP	7.34 ± 1.20	7.4 ± 1.24	0.0560	0.162	.872	NS
IC WIDTH	40.14 ± 1.84	39.58 ± 1.58	0.5560	1.145	.258	NS
TW	52.92 ± 2.83	48.84 ± 3.23	4.0720	4.742	<0.001	S^***^
SW	68.02 ± 3.59	64.32 ± 4.08	3.708	3.410	.001	S^**^
SI	9.47 ± 1.38	8.93 ± 1.63	5.34	1.249	0.22	NS
BC_L	14.1 ± 1.72	12.82 ± 2.30	1.2760	2.219	.032	S^*^
BC_R	13.44 ± 2.18	11.86 ± 1.85	1.5840	2.768	.008	S^*^
PC_L	7.49 ± 1.55	7.69 ± 1.77	0.2000	0.425	.672	NS
PC_R	7.18 ± 1.54	6.93 ± 1.45	.2440	0.576	.567	NS
BC RATIO	5.91 ± 3.2	6.17 ± 3.85	2.62	−2.62	0.012	S^*^
PC RATIO	7.78 ± 3.36	7.61 ± 5.09	1.76	1.45	.155	NS
U VERT	6.92 ± 1.61	6.87 ± 1.61	.0520	0.114	.910	NS
L VERT	11.72 ± 1.63	10.42 ± 1.23	1.3000	3.18	.003	S^**^

*NS p* > 0.05: insignificant

^*^ *p* < 0.05, significant at 5 % significance level; ^**^ *p* < 0.01, significant at 1 % significance level; ^***^ *p* < 0.001, highly significant

**Table 4:** Comparison of male and female in vertical growth pattern (comparison of groups 5 and 6)

Parameter	Males	Females	Difference	*t* value	*p* value	Significance
	Mean ± SD	Mean ± SD				
U1 EXP.	10.42 ± 1.28	9.54 ± .93	0.88	2.78	.008	S^**^
LL-U1	1.82 ± 2.02	1.23 ± 1.25	0.59	1.25	.219	NS
IL GAP	12.95 ± 2.79	11.25 ± 2.03	1.70	2.46	.017	S^*^
IC WIDTH	39.81 ± 1.85	38.52 ± 2.32	1.29	2.17	.035	S^*^
TW	54.53 ± 4.22	50.55 ± 3.87	3.98	3.47	.001	S^**^
SW	65.31 ± 5.24	64.94 ± 5.58	3.72	.24	.809	NS
SI	5.49 ± 1.98	5.91 ± 9.19	4.16	0.96	0.344	NS
BC_L	12.72 ± 2.39	13.04 ± 2.28	0.320	−.49	.63	NS
BC_R	12.39 ± 2.17	12.39 ± 2.23	0.000	.00	1.00	NS
PC_L	5.51 ± 1.63	6.44 ± 1.22	0.924	−2.27	.028	S^*^
PC_R	5.19 ± 1.70	6.24 ± 1.42	1.05	−2.37	.022	S^*^
BC RATIO	7.75 ± 8.19	5.96 ± 3.9	1.798	1.09	.278	NS
PC RATIO	1.25 ± 2.05	7.80 ± 4.38	4.678	1.14	.260	NS
U VERT	6.81 ± 1.47	6.32 ± 1.34	0.488	1.23	.226	NS
L VERT	11.23 ± 1.59	9.89 ± 2.05	1.34	2.59	.013	S^*^

*NS p* > 0.05: insignificant

^*^ *p* < 0.05, significant at 5 % significance level; ^**^ *p* < 0.01, significant at 1 % significance level

As seen in Table 2, lower lip to maxillary incisor distance, interlabial gap, total width of visible teeth, posterior corridor ratio, and lower vertical lip thickness were significantly higher in males as compared to females in average growth pattern group.

According to Table 3, total width of visible teeth, smile width, buccal corridors left and right and lower vertical lip thickness were significantly higher in males as compared to females in horizontal growth pattern group.

Table 4 shows upper incisor exposure, interlabial gap, inter canine width, total width of visible teeth, posterior corridors left and right, and lower vertical lip thickness were significantly higher in males as compared to females in vertical growth pattern group.

Comparison of males in average group with horizontal growth pattern group and vertical growth pattern group was done separately as shown in Tables 5 and 6, respectively. Also comparison of females in average growth pattern group with horizontal growth pattern group and vertical growth pattern group was done separately as shown in Tables 7 and 8, respectively.

**Table 5:** Comparison of males between average and horizontal facial growth pattern (comparison of groups 1 and 3)

Parameter	Average (*n* = 25)	Horizontal (*n* = 25)	Difference	*t* value	*p* value	Significance
U1 EXP.	8.65 ± 1.04	6.93 ± 1.04	1.72	5.933	0.000	S^***^
LL-U1	0.80 ± 0.97	0.30 ± 0.62	0.50	2.168	0.035	S^*^
IL GAP	9.71 ± 1.64	7.34 ± 1.20	2.36	5.814	0.000	S^***^
IC WIDTH	40.29 ± 2.39	40.14 ± 1.85	0.14	0.245	0.807	NS
TW	53.43 ± 3.88	52.92 ± 2.83	0.50	0.529	0.599	NS
SW	67.39 ± 4.63	68.02 ± 3.59	0.63	0.539	0.592	NS
SI	7.16 ± 1.50	9.47 ± 1.38	2.30	5.646	0.000	S^***^
BC_L	13.90 ± 1.54	14.10 ± 1.72	0.20	0.441	0.661	NS
BC_R	13.66 ± 2.29	13.44 ± 2.18	0.21	0.342	0.734	NS
PC_L	7.60 ± 2.38	7.50 ± 1.55	0.10	0.176	0.861	NS
PC_R	6.82 ± 2.15	7.18 ± 1.54	0.35	0.672	0.505	NS
BC RATIO	0.60 ± 0.052	0.59 ± 0.032	0.009	0.752	0.456	NS
PC RATIO	0.795 ± 0.067	0.78 ± 0.034	0.016	1.108	0.274	NS
U VERT	5.98 ± 1.27	6.92 ± 1.61	0.944	2.301	0.026	S^*^
L VERT	11.20 ± 1.03	11.72 ± 1.63	0.52	1.367	0.178	NS

*NS p* > 0.05: insignificant

^*^ *p* < 0.05, significant at 5 % significance level; ^***^ *p* < 0.001, highly significant

**Table 6:** Comparison of males between average and vertical facial growth pattern (comparison of groups 1 and 5)

Parameter	Average (*n* = 25)	Vertical (*n* = 25)	Difference	*t* value	*p* value	Significance
U1 EXP.	8.65 ± 1.04	10.42 ± 1.28	1.77	5.36	0.000	S^***^
LL-U1	0.80 ± 0.97	1.82 ± 2.02	1.02	2.28	0.027	S^*^
IL GAP	9.71 ± 1.64	12.94 ± 2.79	3.24	4.99	0.000	S^***^
IC WIDTH	40.29 ± 2.39	39.80 ± 1.85	0.48	0.79	0.431	NS
TW	53.43 ± 3.88	54.52 ± 4.21	1.10	0.96	0.342	NS
SW	67.39 ± 4.63	65.31 ± 5.23	2.08	1.49	0.143	NS
SI	7.16 ± 1.50	5.49 ± 1.97	1.66	3.36	0.002	S^**^
BC_L	13.90 ± 1.54	12.72 ± 2.38	1.17	2.07	0.044	S^*^
BC_R	13.66 ± 2.29	12.39 ± 2.17	1.26	2.01	0.049	S^*^
PC_L	7.60 ± 2.38	5.51 ± 1.62	2.08	3.61	0.001	S^**^
PC_R	6.82 ± 2.15	5.18 ± 1.70	1.63	2.97	0.005	S^**^
BC RATIO	0.60 ± 0.052	0.77 ± 0.81	0.175	1.07	0.291	NS
PC RATIO	0.795 ± 0.067	1.24 ± 2.05	0.452	1.10	0.276	NS
U VERT	5.98 ± 1.27	6.81 ± 1.47	0.83	2.14	0.037	S^*^
L VERT	11.20 ± 1.03	11.23 ± 1.58	0.036	0.95	0.925	NS

*NS p* > 0.05: insignificant

^*^ *p* < 0.05, significant at 5 % significance level; ^**^ *p* < 0.01, significant at 1 % significance level; ^***^ *p* < 0.001, highly significant

**Table 7:** Comparison of females between average and horizontal facial growth patterns (comparison of groups 2 and 4)

Parameter	Average (*n* = 25)	Horizontal (*n* = 25)	Difference	*t* value	*p* value	Significance
U1 EXP.	8.45 ± 0.84	7.36 ± 1.14	1.09	3.84	0.000	S^***^
LL-U1	0.14 ± 0.22	0.15 ± 0.29	0.012	0.16	0.870	NS
IL GAP	8.43 ± 0.82	7.40 ± 1.24	1.04	3.47	0.001	S^**^
IC WIDTH	39.33 ± 1.94	39.58 ± 1.57	0.24	0.49	0.623	NS
TW	49.66 ± 3.22	48.84 ± 3.22	0.81	0.89	0.378	NS
SW	65.76 ± 3.41	64.32 ± 4.08	1.44	1.36	0.181	NS
SI	7.86 ± 0.88	8.93 ± 1.62	1.07	2.88	0.006	S^**^
BC_L	13.21 ± 1.97	12.82 ± 2.30	0.39	6.46	0.521	NS
BC_R	12.68 ± 2.01	11.86 ± 1.85	0.82	1.50	0.140	NS
PC_L	7.98 ± 2.24	7.69 ± 1.76	0.29	0.51	0.611	NS
PC_R	7.41 1.96	6.93 ± 1.45	0.48	0.99	0.325	NS
BC RATIO	0.59 ± 0.04	0.61 ± 0.038	0.017	1.57	0.122	NS
PC RATIO	0.76 ± 0.06	0.76 ± 0.050	0.004	0.257	0.798	NS
U VERT	5.96 ± 1.50	6.87 ± 1.62	0.90	2.05	0.046	S^*^
L VERT	10.33 ± 1.08	10.42 ± 1.22	0.092	0.28	0.780	NS

*NS p* > 0.05: insignificant

^*^ *p* < 0.05, significant at 5 % significance level; ^**^ *p* < 0.01, significant at 1 % significance level; ^***^ *p* < 0.001, highly significant

**Table 8:** Comparison of females having average and vertical facial growth pattern (comparison of groups 2 and 6)

Parameter	Average (*n* = 25)	Vertical (*n* = 25)	Difference	*t* value	*p* value	Significance
U1 EXP.	8.45 ± 0.84	9.53 ± 0.93	1.08	4.28	0.000	S^***^
LL-U1	0.14 ± 0.22	1.23 ± 1.24	1.09	4.31	0.000	S^***^
IL GAP	8.43 ± 0.82	11.24 ± 2.02	2.81	6.42	0.000	S^***^
IC WIDTH	39.33 ± 1.94	38.52 ± 2.32	0.82	1.35	0.185	NS
TW	49.66 ± 3.22	50.55 ± 3.87	0.89	0.88	0.381	NS
SW	65.76 ± 3.41	64.94 ± 5.58	0.82	0.63	0.534	NS
SI	7.86 ± 0.88	5.90 ± 0.92	1.96	7.66	0.000	S^***^
BC_L	13.21 ± 1.97	13.04 ± 2.27	0.177	0.29	0.772	NS
BC_R	12.68 ± 2.01	12.39 ± 2.22	0.288	0.48	0.634	NS
PC_L	7.98 ± 2.24	6.43 ± 1.21	1.55	3.04	0.004	S^**^
PC_R	7.41 1.96	6.23 ± 1.41	1.18	2.45	0.018	S^*^
BC RATIO	0.59 ± 0.04	0.59 ± 0.03	0.004	0.36	0.723	NS
PC RATIO	0.76 ± 0.06	0.78 ± 0.04	0.023	1.55	0.127	NS
U VERT	5.96 ± 1.50	6.32 ± 1.33	0.356	0.89	0.380	NS
L VERT	10.33 ± 1.08	9.88 ± 2.04	0.444	0.96	0.343	NS

*NS p* > 0.05: insignificant

^*^ *p* < 0.05, significant at 5 % significance level; ^**^ *p* < 0.01, significant at 1 % significance level; ^***^ *p* < 0.001, highly significant

According to Table 5, upper incisor exposure and interlabial gap were significantly higher in males of average growth pattern group as compared to males of horizontal growth pattern group. Upper vertical lip thickness was significantly higher in males of horizontal growth pattern group.

As seen in Table 6, upper incisor exposure, upper incisor to lower lip distance, interlabial gap, and upper vertical lip thickness were significantly higher in males of vertical growth pattern group as compared to males of average growth pattern group. Buccal corridors left and right and posterior corridors left and right were higher in average growth pattern group.

Table 7 shows upper incisor exposure and interlabial gap were significantly higher in females of average growth pattern group as compared to females of horizontal growth pattern group. Upper vertical lip thickness was significantly higher in females of horizontal growth pattern group.

As shown in Table 8, upper incisor exposure, upper incisor to lower lip distance, and interlabial gap were significantly higher in females of vertical growth pattern group as compared to females of average growth pattern group. Posterior corridors left and right were higher in average growth pattern group.

These results clearly show that the vertical parameters of smile were highest in vertical growth pattern group and least in horizontal growth pattern group in both males and females.

Correlation between smile parameters and skeletal parameters of males in average facial growth pattern group, females in average facial growth pattern group, males in horizontal group, females in horizontal growth pattern group, males in vertical growth pattern group, and females in vertical growth pattern group is in Additional file 1.

Interlabial gap, when correlated with cephalometric parameters, was found to be positively correlated with overjet in average facial growth pattern group of males and was positively correlated with posterior facial height in average facial growth pattern group of females. The interlabial gap was positively correlated with lower anterior facial height in males having horizontal facial growth pattern and no correlations with other cephalometric measurements in females having horizontal facial growth pattern. In the vertical facial growth pattern group, interlabial gap was positively correlated with Y-axis and lower vertical lip length in males but insignificant correlations amongst females.

The incisal edge of the maxillary incisor to lower lip distance was found to be positively correlated with mandibular plane angle in average facial growth pattern group of males and was positively correlated with overjet in average facial growth pattern group of females. It was also positively correlated with palatal plane angle of females in horizontal facial growth pattern group.

Based on these tables, it can be inferred that saddle angle and lower incisor to NB (angular and linear) was not found to be related to smile parameters and also upper incisor to NA (angular) was found to be positively correlated with upper lip vertical in females of all the three groups.

## Discussion

To study a smile beyond static pictures, capturing a dynamic smile [2, 7, 10] was used, thus avoiding the inherent error of a single snapshot. The variables of smile were significantly affected by the facial growth pattern in this study.

### Upper incisor exposure

The upper incisor exposure was less in females when compared with males in all three groups and this difference was significant in vertical facial growth pattern group. This is contrary to the findings of Vig and Brundo [11], Peck et al. [12, 13], and Balani et al [14], whereas the above finding is supported by a study done by Weeden et al. [15], where the results demonstrated that males exhibited greater amount of facial movements than females thus increasing the incisal display on smiling.

### Incisal display

The incisal display significantly increased from horizontal to average to vertical facial growth pattern, with least incisal display in horizontal facial growth pattern subjects and maximum in vertical facial growth pattern subjects for both males and females. Contrary to this, Mc Namara et al. [16] found that the vertical display on smile of the maxillary right central incisor could not be correlated with the skeletal vertical dimension, as measured from nasion to menton and from anterior nasal spine to menton.

On correlating upper incisor exposure with cephalometric parameters, it was found that the upper incisor display was negatively correlated with posterior facial height and Jaraback’s ratio of males in horizontal facial growth pattern group but not in females and it was positively correlated with overjet, Y-axis, and palatal plane angle of males in vertical facial growth pattern group.

### Incisal edge to lower lip distance

The incisal edge to lower lip distance was less in females when compared with males in all three groups and this difference was significant in average facial growth pattern group. This is supported by the findings of Vig and Brundo [11] and Peck et al. [12, 13] who found less mandibular tooth exposure in females than males at all ages.

The distance between incisal edge of the maxillary incisor and lower lip was least in horizontal growers and the maximum in vertical growers in both male and female.

No correlations of distance between incisal edge of the maxillary incisor and lower lip were found with cephalomteric measurements in the vertical facial growth pattern group.

### Interlabial gap

The interlabial gap was significantly more in males when compared to that in females in average and vertical facial growth pattern group. This was contrary to Rigsbee et al. [17], Tjan et al. [1], and Jensen et al [18] and is supported by a study done by Weeden et al. [15], where the results demonstrated that males exhibited greater amount of facial movements than females thus increasing the interlabial gap on smiling.

Interlabial gap was significantly found to be maximum in vertical growers, followed by average and least in horizontal growers in both male and female.

### Intercanine width

The intercanine width was more in males when compared to that in females in all groups but this difference was statistically significant only in the vertical facial growth pattern group.

Intercanine width was positively correlated with total width of visible maxillary teeth on smile of males in average and vertical facial growth pattern groups but not of males in horizontal growth pattern group. In males and females of vertical growth pattern group, the intercanine width was positively correlated with smile width and total width of visible maxillary teeth on smile.

The intercanine width was found to be least in vertical growers when compared with horizontal and average growers in both male a females. Similar results were found in a study done by Grippaudo et al. [19], in skeletal class II subjects. Changes in upper arch shape with intercanine diameter were proportionately smaller in patients with high angles and larger in low-angle patients.

### Total width of all visible maxillary teeth

The total width of all visible maxillary teeth was significantly more in males when compared to that in females in all groups.

The total width of all visible maxillary teeth was negatively correlated with posterior corridor (left and right side) in all the groups indicating that in case of increased visible teeth during smile, the posterior corridor decreased. Due to the same reason, the total width of all visible maxillary teeth was negatively correlated with posterior corridor ratio in all the groups.

### Smile width

The smile width was more in males when compared to that in females in all groups, but this difference was statistically significant only in the horizontal facial growth pattern group. This was contrary to the results of Rigsbee et al. [17] and Chetan et al. [20] who found that females exhibited more animation as compared to men resulting from a greater degree of upper lip elevation and increased width resulting in an increased display of teeth.

On comparing smile width separately in horizontal and vertical facial growth pattern group with average facial growth pattern group, statistically insignificant difference was found in both males and females in both the facial growth pattern groups.

### Buccal corridor

With regard to the buccal corridor of males when compared with females, the mean value was found to be more in case of males in all groups but was significantly more only in males in horizontal facial growth pattern group. A similar finding was reported by Maulik and Nanda [21]who found that females had less buccal corridor space than males.

As regards buccal corridor of the left side when compared with the right side, statistically significant difference was found which demonstrates that the buccal corridor was greater on the left side than on the right side. This finding is supported by the study done by Okamoto et al. [22] where it was found that the displacements of the right and left corners of the mouth during voluntary smile were asymmetric and the left-sided laterality was found.

Buccal corridor of the left side when compared with the right side, in vertical facial growth pattern group with average facial growth pattern group, was found to be significantly less in vertical facial growth pattern group in males but not in females. The above findings are supported by the results of Yang et al. [23] who found that FMA and LAFH were negatively correlated with buccal corridor.

The buccal corridors (left and right) were positively correlated to each other indicating that if the buccal corridor was increased on the left side, it would increase on the right side as well, in all the groups irrespective of the age. A similar finding was observed by Krishnan et al. [6] showing a high correlation between the right and left buccal corridor spaces in both men and women. It was also positively correlated with posterior corridor (left and right) indicating that in case of increased buccal corridor, the posterior corridor also increased. No correlation was found between the total anterior facial height and the buccal corridor space similar to the findings of Yang et al [23].

### Posterior corridor

Posterior corridor of males, when compared with females, was found to be less in case of males in horizontal and average facial growth pattern groups but was statistically significantly less in males in vertical facial growth pattern group. On comparing posterior corridor of the left and right sides, statistically significant difference was found which indicate that the posterior corridor was greater on the left side than on the right side. This finding is supported by the study done by Okamoto et al. [22] where it was found that the displacements of the right and left corners of the mouth during voluntary smile were asymmetric, and the left-sided laterality was found.

Posterior corridor in vertical facial growth pattern group, when compared with average facial growth pattern group, was found to be significantly less in vertical facial growth pattern group in both males and females. On comparing buccal corridor of left and right sides in horizontal facial growth pattern group with average facial growth pattern group, it was found to be insignificantly different in both males and females.

The posterior corridors (left and right) were positively correlated to each other indicating that if the posterior corridor was increased on the left side, it would increase on the right side as well, in all the groups irrespective of the age.

### Upper lip vertical

Upper lip vertical length in males and females demonstrated an insignificant difference in all the groups.

Upper lip vertical length of horizontal facial growth pattern group, when compared with average facial growth pattern group, indicated that upper lip vertical length was more in horizontal and vertical growth pattern group in both males and females and least in average growers.

### Lower lip vertical

For lower lip vertical length in males when compared with females, statistically significant difference was found showing increased lower vertical lip length in males in all the three groups.

With regard to the lower lip vertical length in horizontal and vertical facial growth pattern group when compared with average facial growth pattern group, no significant difference was found between the two groups in both males and females. In a study done by Joshi et al. [24], the lip position in relation to various malocclusions was studied which showed a significant difference in the sagittal lip positions in different skeletal malocclusions. Thus, it can be inferred vertical as well as the sagittal skeletal features influence the overall soft tissue drape.

To homogenize the sample, only class I subjects were selected. Increasing the sample size randomly followed by categorizing them based on Angle’s classification and using regression model to compute additional variables could further improve the study. The use of a three-dimensional methodology [25] can be used for analyzing anthropometric characteristics of soft tissue of face.

## Conclusions

From the analysis and obtained results, the following conclusions can be drawn:

1. Smile parameters in males and females were statistically significantly different, with higher mean values for upper incisor exposure, incisal edge to lower lip distance, interlabial gap, intercanine width, total width, smile width, and lower lip vertical length in males than in females.
2. For the horizontal facial growth pattern group when compared to average facial growth pattern group, statistically significant higher mean differences were observed in average growth pattern in upper incisor exposure, incisal edge to lower lip distance, interlabial gap, and upper vertical lip length, irrespective of the sex. The smile index was more and difference was statistically significant in horizontal facial growth pattern group, in both males and females.
3. Vertical facial growth pattern group exhibited statistically significant higher mean difference in upper incisor exposure, incisal edge to lower lip distance, interlabial gap, upper vertical lip length, and occlusal plane angle, than average facial growth pattern group in both males and females; whereas, the smile index and posterior corridor (left and right) were statistically significantly less in vertical facial growth pattern group in both males and females.
4. Intercanine width exhibited positive correlation with total width of all visible teeth during smile in average facial growth pattern group of males and in vertical facial growth pattern group of both males and females.
5. Smile width was positively correlated with posterior corridor (left and right) and buccal corridor (left and right) in all the groups in both males and females.
6. Buccal corridor of the left side was positively correlated with buccal corridor of the right side and posterior corridor of both sides in all the groups in both males and females.
7. Posterior corridor of the left side was positively correlated with posterior corridor of the right side in all the groups in both males and females.
8. Posterior corridor ratio was positively correlated with buccal corridor ratio in all the groups in both males and females.
9. Saddle angle and lower incisor to NB (angular and linear) were not found to be related to smile.
10. Upper incisor to NA (angular) was found to be positively correlated with upper lip vertical in females of all the three groups.

## Additional file

Additional file 1:
**Correlation between smile parameters and skeletal parameters of males and females in different groups.** Correlation between smile parameters and skeletal parameters of males in average facial growth pattern group, females in average facial growth pattern group, males in horizontal group, females in horizontal growth pattern group, males in vertical growth pattern group, and females in vertical growth pattern group.
