# Supplementary material for: Smile analysis in different facial patterns and its correlation with underlying hard tissues
Source: Prog Orthod. 2015 Sep 4;16:28. doi: 10.1186/s40510-015-0099-4 (PMC4560731; doi:10.1186/s40510-015-0099-4)
Supplement: Additional file 1: — Correlation between smile parameters and skeletal parameters of males and females in different groups. Correlation between smile parameters and skeletal parameters of males in average facial growth pattern group, females in average facial growth pattern group, males in horizontal group, females in horizontal growth pattern group, males in vertical growth pattern group, and females in vertical growth pattern group. [file 40510_2015_99_MOESM1_ESM.doc]

**Additional file 1**

1. CORRELATION BETWEEN PARAMETERS OF SMILE OF MALES IN AVERAGE GROWTH PATTERN GROUP

**
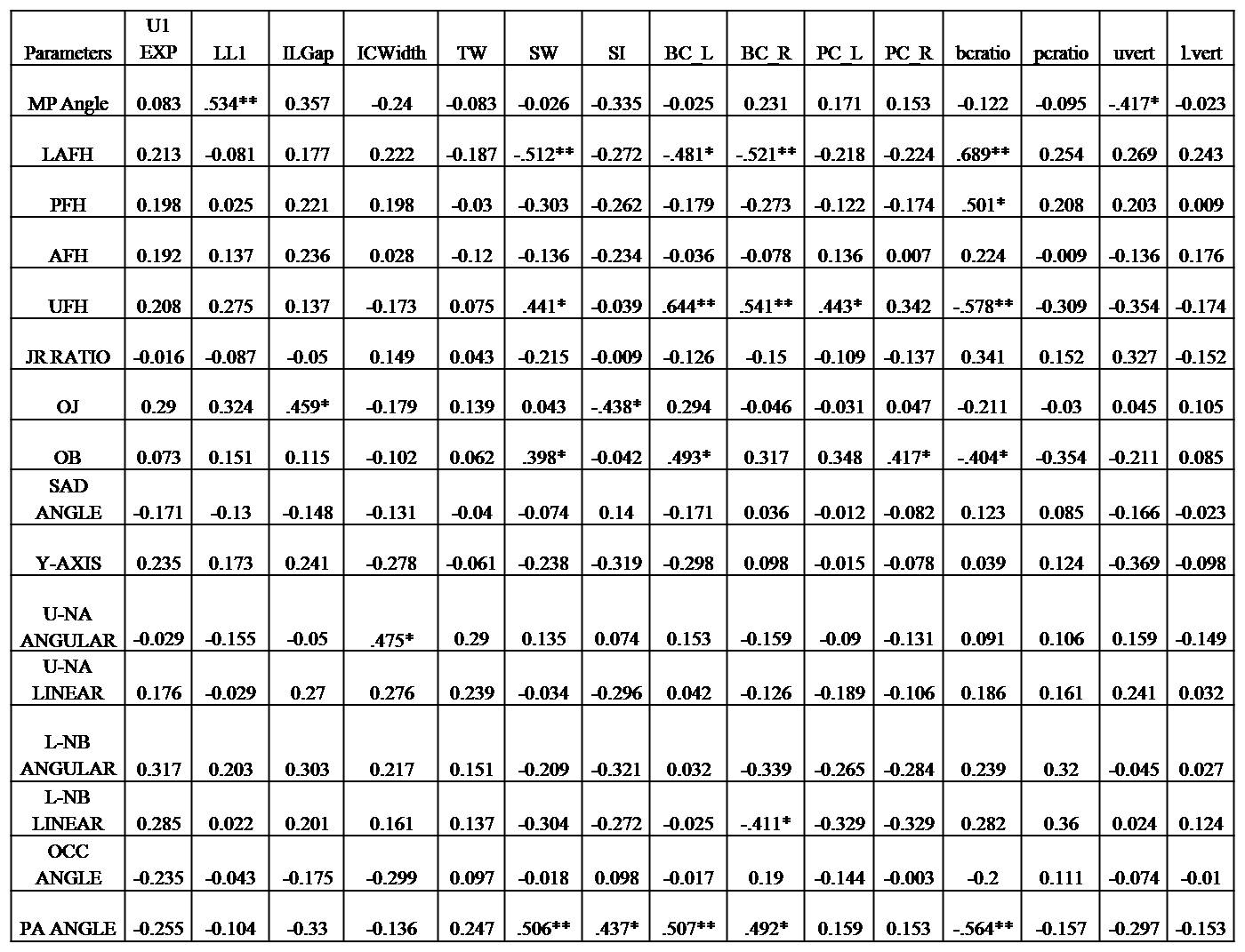
**

| **. Correlation is significant at the 0.01 level |
| --- |
| *. Correlation is significant at the 0.05 level |

1. CORRELATION BETWEEN SMILE AND CEPHALOMETRIC PARAMETERS OF FEMALES IN AVERAGE GROWTH PATTERN GROUP

**
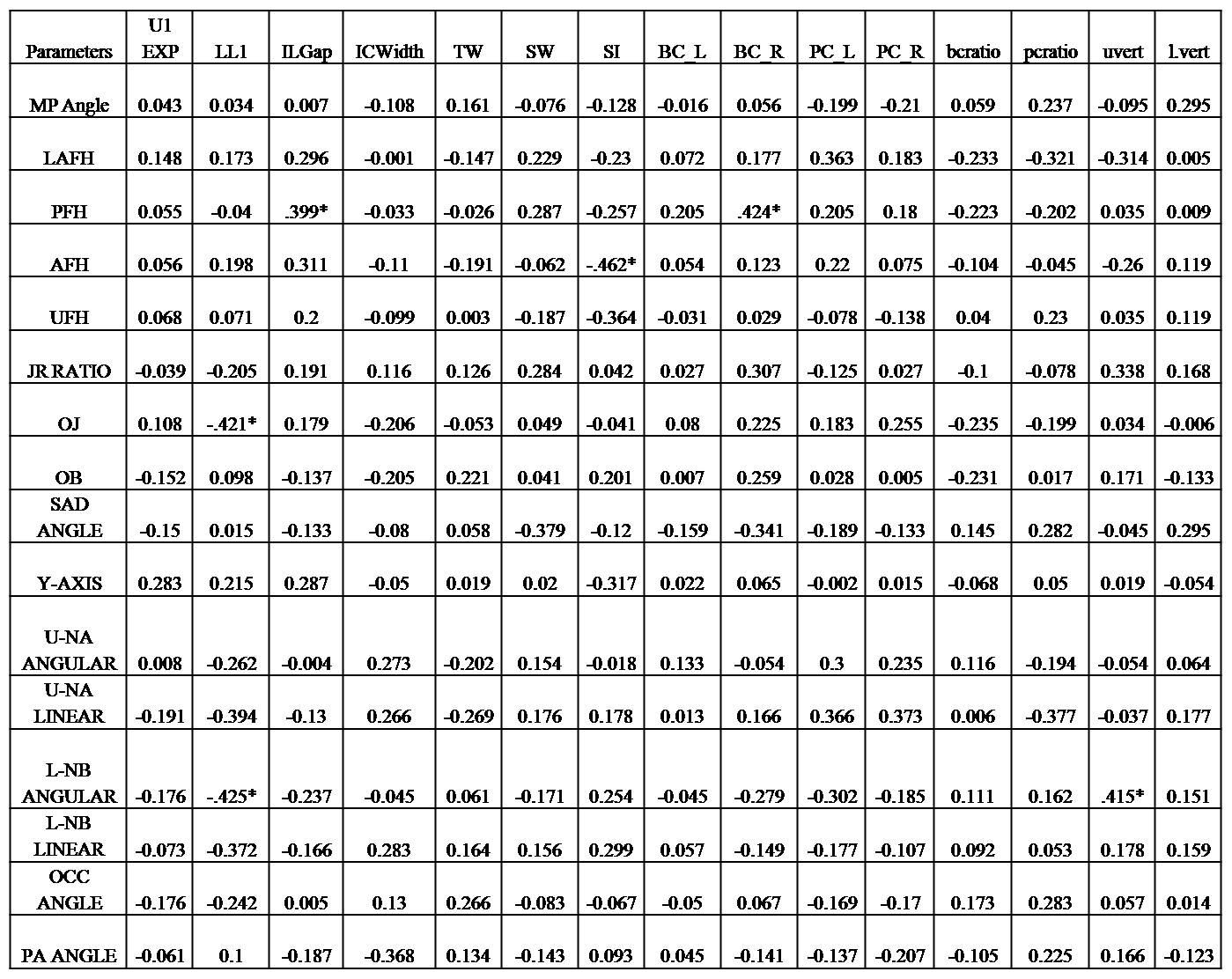
**

| **. Correlation is significant at the 0.01 level |
| --- |
| *. Correlation is significant at the 0.05 level |

1. **0CORRELATION BETWEEN SMILE AND CEPHALOMETRIC PARAMETERS OF MALES IN HORIZONTAL GROWTH PATTERN GROUP**

| 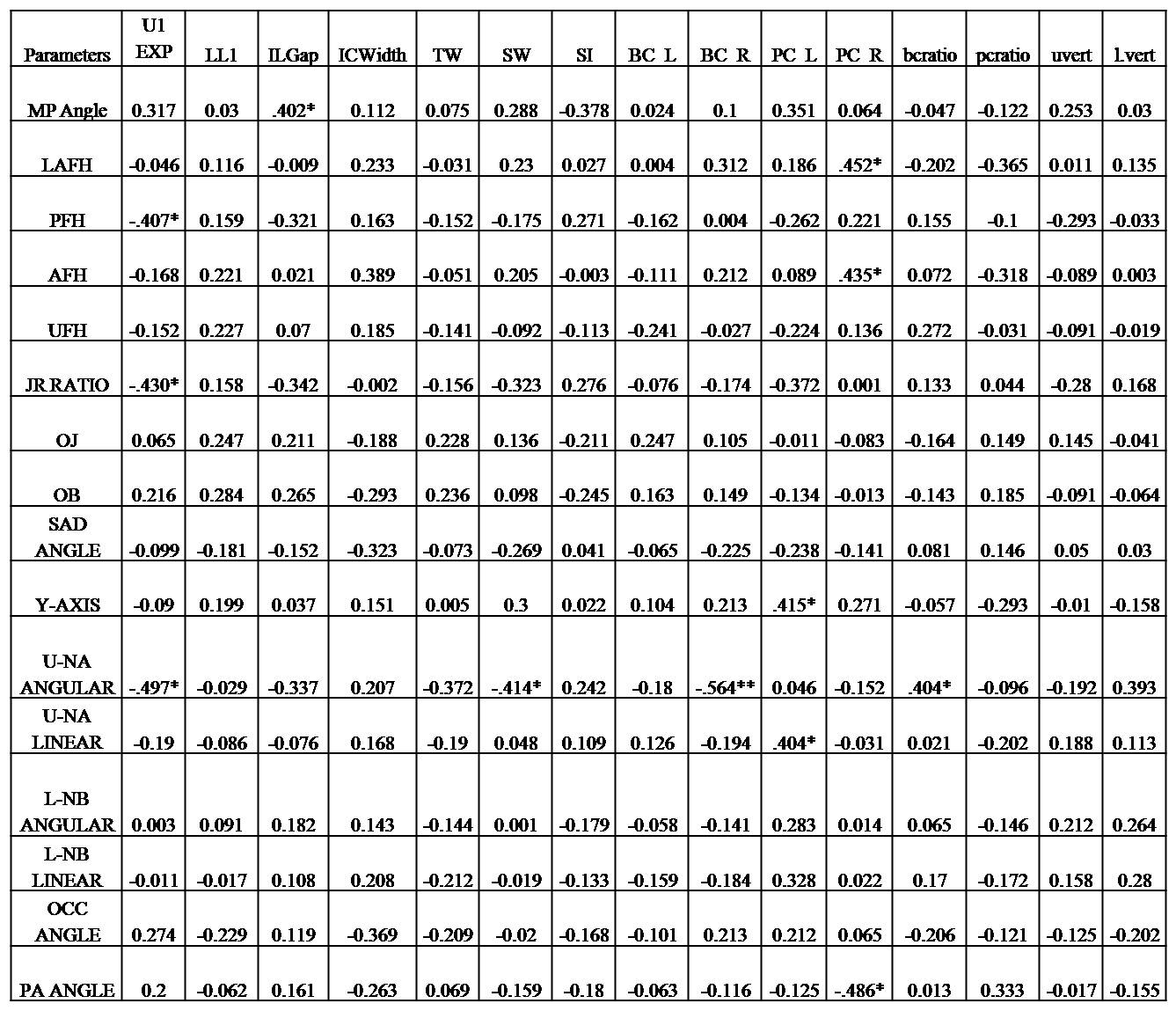  **. Correlation is significant at the 0.01 level |
| --- |
| *. Correlation is significant at the 0.05 level |

1. CORRELATION BETWEEN SMILE AND CEPHALOMETRIC PARAMETERS OF FEMALES IN HORIZONTAL GROWTH PATTERN GROUP


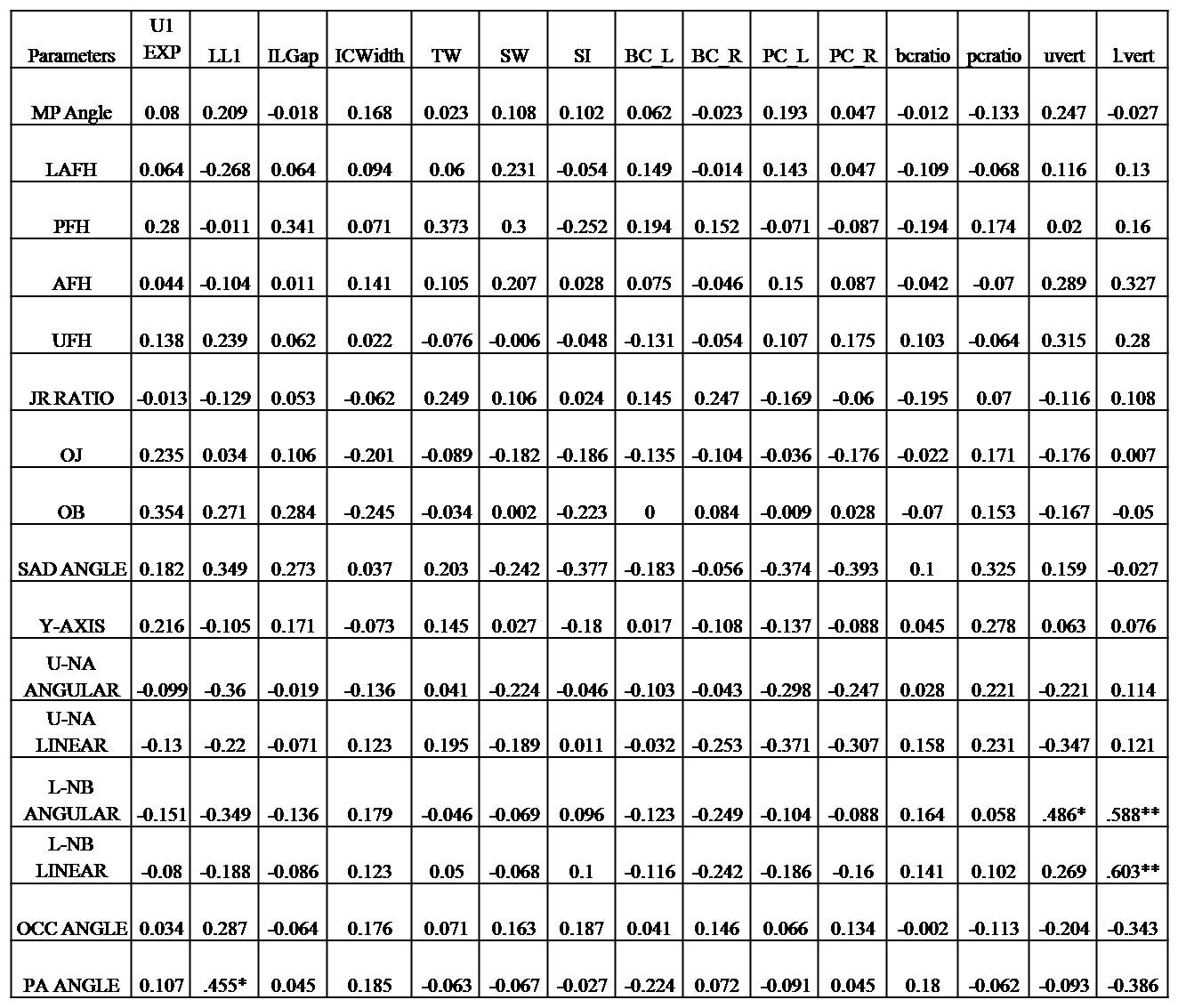


| **. Correlation is significant at the 0.01 level |
| --- |
| *. Correlation is significant at the 0.05 level |

1. CORRELATION BETWEEN SMILE AND CEPHALOMETRIC PARAMETERS OF MALES IN VERTICAL GROWTH PATTERN GROUP

**
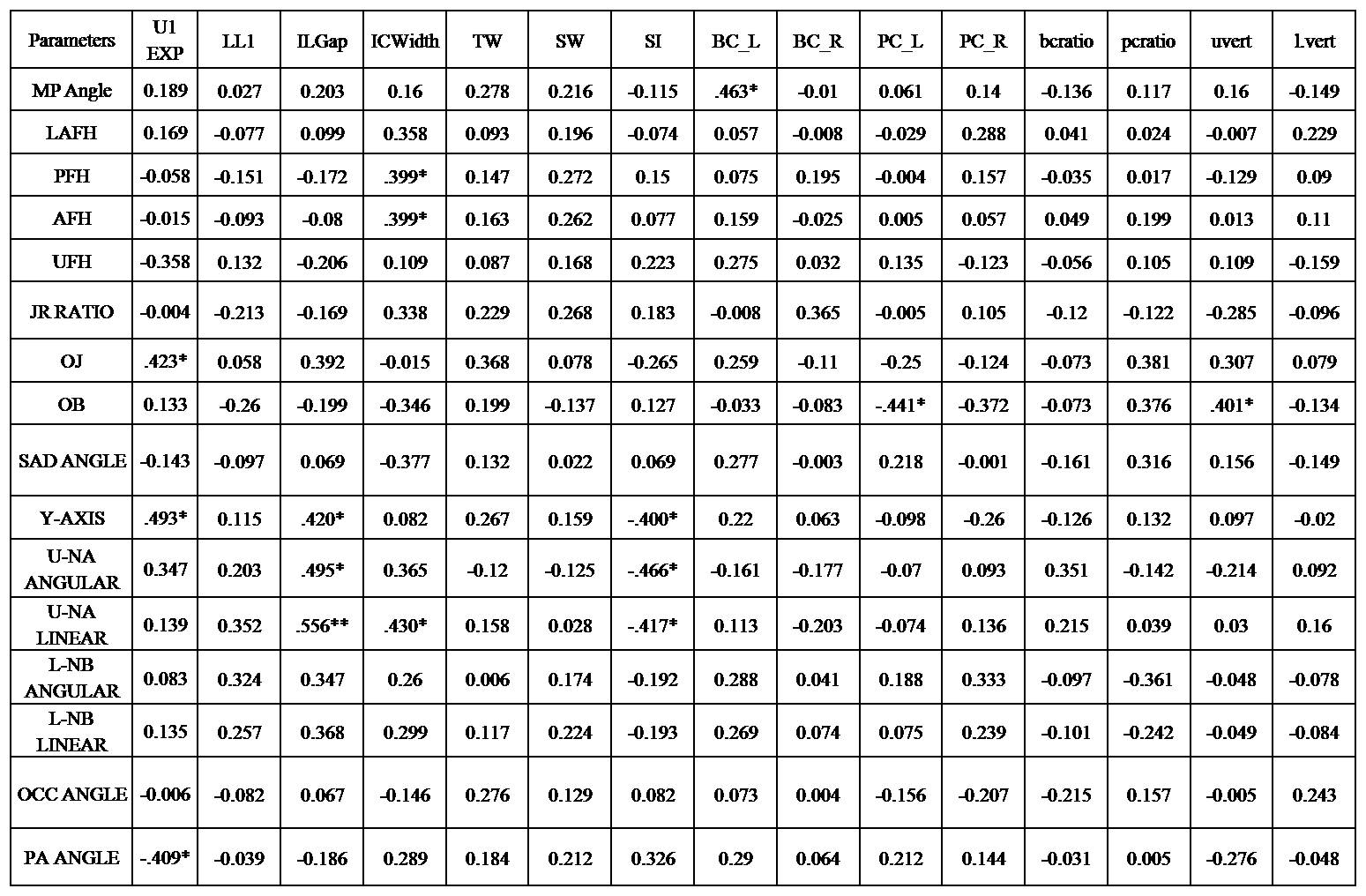
**

| **. Correlation is significant at the 0.01 level |
| --- |
| *. Correlation is significant at the 0.05 level  0 |

1. CORRELATION BETWEEN SMILE AND CEPHALOMETRIC PARAMETERS OF FEMALES IN VERTICAL GROWTH PATTERN GROUP


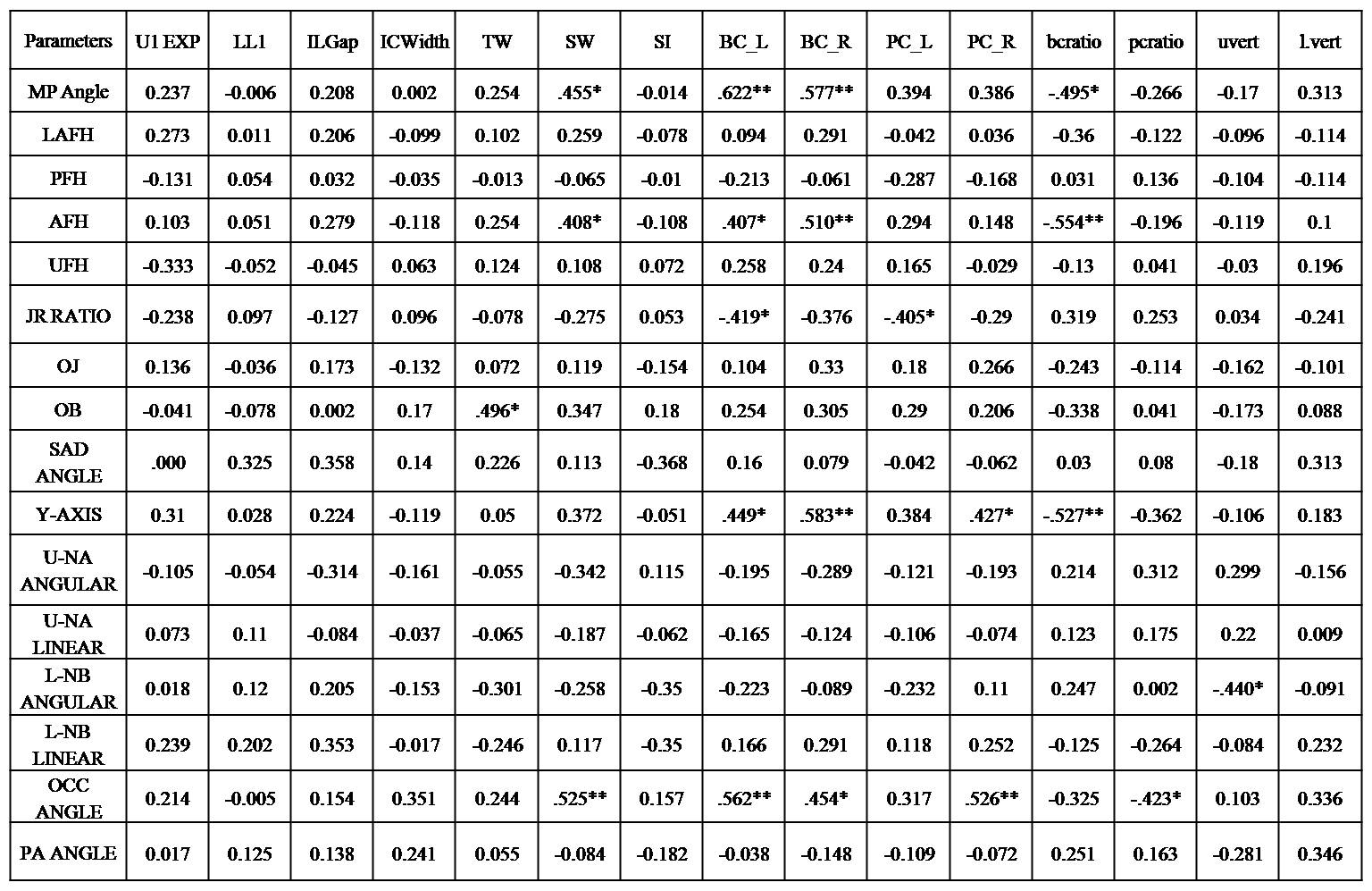


| **. Correlation is significant at the 0.01 level  *. Correlation is significant at the 0.05 level |
| --- |
